# Draft Genome Sequence of Mycobacterium montefiorense Isolated from Japanese Black Salamander (Hynobius nigrescens)

**DOI:** 10.1128/genomeA.00448-18

**Published:** 2018-05-24

**Authors:** Hanako Fukano, Mitsunori Yoshida, Akane Shimizu, Hajime Iwao, Yukie Katayama, Tsutomu Omatsu, Tetsuya Mizutani, Osamu Kurata, Shinpei Wada, Yoshihiko Hoshino

**Affiliations:** a Department of Mycobacteriology, Leprosy Research Center, National Institute of Infectious Diseases, Tokyo, Japan; b The Laboratory of Aquatic Medicine, Nippon Veterinary and Life Science University, Tokyo, Japan; c Niigata City Aquarium, Niigata, Japan; d Research and Education Center for Prevention of Global Infectious Diseases of Animals, Tokyo University of Agriculture and Technology, Tokyo, Japan

## Abstract

Mycobacterium montefiorense is a member of the Mycobacterium simiae complex, the largest group of nontuberculous mycobacteria. Here, we report the genome sequence of M. montefiorense isolate BS, isolated from diseased Japanese black salamander (Hynobius nigrescens) reared in an aquarium in Japan. This is the first reported case of an M. montefiorense infection in an amphibian.

## GENOME ANNOUNCEMENT

Nontuberculous mycobacteria (NTMs) are ubiquitous in the environment and exist even in water (1). Some of the species can cause outbreaks of NTM infectious disease in humans or fish (2–4). Mycobacterium montefiorense is a member of the M. simiae complex, which is the largest group of nontuberculous mycobacterial species (5). In 2003, this species was reported as a new nontuberculous mycobacterium (NTM) species closely related to M. triplex, a human-pathogenic species (6). It was first isolated from a disease outbreak in two species of moray eels, green moray (Gymnothorax funebris) and spotted moray (G. moringa), caught near the Florida Keys in 1991 to 1992 and being reared in synthetic seawater for an exhibit (7). This captive population of moray eels died from this NTM infection while showing ulcerative granulomatous dermatitis and fasciitis (7).

From 2010 to 2014, an outbreak of infections caused by M. montefiorense was recorded in some salamander species, namely, Japanese black salamander (Hynobius nigrescens) and Hakuba salamander (H. hidamontanus) reared in an aquarium. Some of the diseased salamanders showed ulcerative lesions on the skin, but the most prominent features were granulomatous lesions observed in the liver with numerous acid-fast bacteria present. We isolated this causative agent from the liver of a dead Japanese black salamander and named the strain BS.

We hereby report the draft genome sequence of M. montefiorense strain BS isolated from Japanese black salamander. This is the first case of an M. montefiorense infection of amphibians.

The strain was inoculated on Middlebrook 7H10 agar supplemented with 10% oleic acid-albumin-dextrose-catalase enrichment and incubated at 25°C for about 1 to 2 months. Genomic DNA was extracted by a phenol-chloroform method.

Libraries were constructed using the Nextera XT DNA library preparation kit (Illumina, Inc., San Diego, CA, USA). The genome sequence was obtained by using Illumina 300-bp × 2 paired-end reads (741,188,100 reads) (Illumina, Inc.). The paired-end reads were assembled with Platanus version 1.1 into 50 contigs. Automated annotation was carried out with the DDBJ FAST Annotation and Submission Tool (DFAST) (https://dfast.nig.ac.jp). Average nucleotide identity (ANI) was calculated with JSpeciesWS version 1.2.1 (8, 9).

The genome of M. montefiorense BS is 5,744,567 bp in length, with a 65.1% G+C content. In total, 5,384 protein-coding sequences, 3 rRNAs, and 53 tRNAs were predicted. The ANI values versus M. simiae ATCC 25275^T^ (GenBank accession numbers CBMJ020000001 to CBMJ020000359) and M. triplex DSM 44626^T^ (GenBank accession number NZ_CCAU000000000) were 85.32% and 81.97%, respectively.

This sequence is the first genome sequence of M. montefiorense isolated from an amphibian, and it will provide significant epidemiological information of NTMs in an aquatic environment.

### Accession number(s).

The scaffold sequences and annotations of M. montefiorense BS were deposited at DDBJ/EMBL/GenBank under the accession number BFCH00000000.
